# The Directed Differentiation of Human iPS Cells into Kidney Podocytes

**DOI:** 10.1371/journal.pone.0046453

**Published:** 2012-09-28

**Authors:** Bi Song, Alexandra M. Smink, Christina V. Jones, Judy M. Callaghan, Stephen D. Firth, Claude A. Bernard, Andrew L. Laslett, Peter G. Kerr, Sharon D. Ricardo

**Affiliations:** 1 Monash Immunology and Stem Cell Laboratories (MISCL), Monash University, Clayton, Victoria, Australia; 2 Monash Micro Imaging, Monash University, Clayton, Victoria, Australia; 3 CSIRO Materials Science and Engineering, Clayton, Victoria, Australia; 4 Department of Anatomy and Developmental Biology, Monash University, Clayton, Victoria, Australia; 5 Department of Medicine, Monash University, Monash Medical Centre, Clayton, Victoria, Australia; Fondazione IRCCS Ospedale Maggiore Policlinico & Fondazione D’Amico per la Ricerca sulle Malattie Renali, Italy

## Abstract

The loss of glomerular podocytes is a key event in the progression of chronic kidney disease resulting in proteinuria and declining function. Podocytes are slow cycling cells that are considered terminally differentiated. Here we provide the first report of the directed differentiation of induced pluripotent stem (iPS) cells to generate kidney cells with podocyte features. The iPS-derived podocytes share a morphological phenotype analogous with cultured human podocytes. Following 10 days of directed differentiation, iPS podocytes had an up-regulated expression of mRNA and protein localization for podocyte markers including synaptopodin, nephrin and Wilm’s tumour protein (WT1), combined with a down-regulation of the stem cell marker OCT3/4. In contrast to human podocytes that become quiescent in culture, iPS-derived cells maintain a proliferative capacity suggestive of a more immature phenotype. The transduction of iPS podocytes with fluorescent labeled-talin that were immunostained with podocin showed a cytoplasmic contractile response to angiotensin II (AII). A permeability assay provided functional evidence of albumin uptake in the cytoplasm of iPS podocytes comparable to human podocytes. Moreover, labeled iPS-derived podocytes were found to integrate into reaggregated metanephric kidney explants where they incorporated into developing glomeruli and co-expressed WT1. This study establishes the differentiation of iPS cells to kidney podocytes that will be useful for screening new treatments, understanding podocyte pathogenesis, and offering possibilities for regenerative medicine.

## Introduction

The epidemic of chronic kidney disease and end-stage renal failure represents a crisis for healthcare world-wide. Given the high morbidity of dialysis, its cost and the shortage of donor kidneys, there is an urgent need for further therapeutic options. Over two-thirds of patients with chronic kidney disease who progress to end-stage renal failure suffer from disorders that originate in the glomerulus, specifically podocyte injury leading to cell loss and proteinuria [1], [2]. Podocytes are highly specialised cells with a complex cytoarchitecture consisting of tertiary foot processes that form the glomerular filtration barrier. The majority of glomerulopathies are a consequence of podocyte injury, resulting in the initiation and progression of fibrosis and impaired renal function [3], [4]. In contrast to the regeneration of tubular epithelial cells that are rapidly repaired by intrinsic, proliferative expansion [5], the replacement of damaged glomerular podocytes remains a challenge. Podocyte precursors are derived from the metanephric mesenchyme during kidney development. Following maturation they establish their complex cell architecture and become highly terminally differentiated with a very limited regenerative capacity [6], [7].

Likewise, primary cultures of human podocytes are difficult to maintain in culture due to their limited capacity to divide. Therefore, the reprogramming of adult cells to generate induced pluripotent stem (iPS) cells [8], [9], [10] with a high proliferative ability and broad differentiation capacity represents a major advance for both preclinical and clinical applications. iPS cells and their progeny will aid in understanding disease pathogenesis, screening new treatments, and offering possibilities for replacement cells to repair and regenerate damaged kidneys. However, due to the complexity of the developmental processes and kidney structure, there have been few successful reports showing differentiation of pluripotent cells to kidney progenitors. Cultured mouse embryonic stem (ES) have been reported to differentiate into intermediate mesoderm [11] and tubular cells [12], [13], [14] *in vitro* that is enhanced by activin A [15] and retinoic acid [11], however without evidence of integration into the structural development of glomeruli. Moreover, using human ES cells we have previously reported the directed differentiation of an enriched population of mesodermal kidney progenitors [16].

Recently we [17], and others [18] reported the successful generation of iPS cells from human kidney cells that are pluripotent and have long-term proliferative ability. Based on our established protocol using the directed differentiation of human ES cells to kidney progenitors and extensive background transcriptional profiling [16] we now report a reliable and efficient method for differentiation of iPS cells into kidney podocyte progenitors. The iPS-derived podocytes share cell morphology similar to primary human podocytes including tertiary cytoplasmic cell processes by scanning electron microscopy (SEM). By day 10 of differentiation the iPS-derived podocytes show protein localisation of the mesodermal and podocyte markers; Wilm’s tumour protein (WT1), paired homeobox gene 2 (Pax-2), nephrin, podocin and synaptopodin. qPCR confirmed that over the time course of directed differentiation, the iPS-derived podocytes show an upregulated expression of podocyte markers concurrent with a down-regulation of the pluripotency gene, OCT3/4. The iPS-derived podocytes have functional characteristics of podocytes in their contractile response to angiotensin II (AII) and endocytosis of albumin, and furthermore they integrate into WT1-positive glomerular aggregates in developing kidneys.

## Results

### Induction of Kidney Podocyte Progenitors from iPS Cells

**Figure 1:**
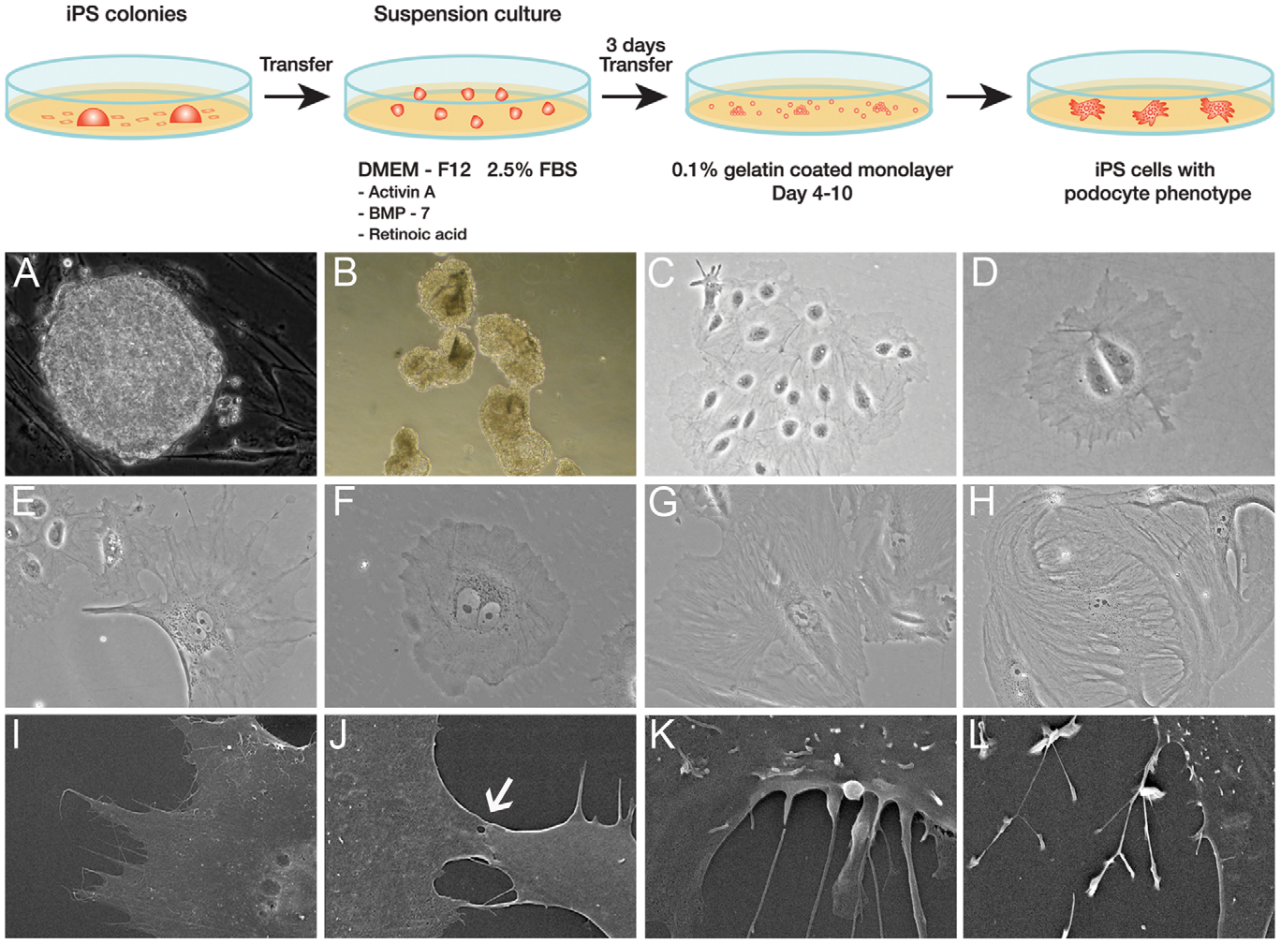
Podocyte Progenitors from iPS cells. Diagrammatic depiction of the directed differentiation of iPS cells to podocyte progenitors (panel). A) iPS colonies derived from human kidney cells as assessed by phase contrast microscopy were B) mechanically dissociated and grown in suspension culture. C) By phase contrast microscopy the cell clusters attached and propagated in differentiation media devoid of a feeder layer. D) Following serial dilution into single cell cultures and 3 days of directed differentiation the cells appeared relatively unspecialised and immature with a rounded and often binucleated phenotype. E) At day 10 of differentiation the iPS-derived cells had cytoplasmic extensions with an arborized appearance resembling podocytes. F) The iPS podocytes were compared to cultured immortalised human podocytes that propagate at a permissive temperature of 33°C. G) At 37°C the immortalised podocytes enter growth arrest and display a typical arborized appearance. H) Primary cultures of human podocytes also contained large multinucleated cells displaying foot process extensions. I) and J) Scanning electron microscopy of iPS podocytes demonstrated a main cell body with cytoplasmic projections and tight junction-like structures connecting adjacent cells (*arrow*). K) Cytoplasmic projections could be observed extending from the cell body of the iPS podocyte. L) Tertiary projections were also common at the end processes of the cytoplasmic projections. Magnification A,B ×200; C,E,G,H ×100; D,F ×400; SEM I ×1.1K, J ×3.5K, K × 8K, L ×2.5K.

The kidney differentiation capacity of iPS cells derived from human mesangial cells [17] was developed as diagrammatically depicted in Figure 1. The iPS colonies that were maintained on mouse embryonic fibroblast (MEFs) feeders exhibited a similar morphology to human ES cells (Figure 1A). Cells were separated from the original cell cluster using serial dilution and compared morphologically to primary human podocytes obtained from patients undergoing nephrectomy. To initiate differentiation, iPS colonies were mechanically cut into pieces that were replated into suspension culture (Figure 1B). Following the addition of activin A, bone morphogenic protein (BMP-7) and retinoic acid, the iPS cells attached to gelatin-coated plates devoid of MEF feeders and were cultured for a further 10 days (Figure 1C). After 10 days, the iPS podocytes were grown in media without the addition of activin A, BMP-7 and retinoic acid where they could be maintained and showed a long-term proliferative capacity.

Putative podocytes differentiated from iPS cells resembled the structural appearance of cultured glomerular podocytes [19], [20]. The progeny of cells grown following serial dilution of the original cluster appeared uniformly unspecialised and immature with a large, rounded and often binucleated phenotype by day 3 after directed differentiation (Figure 1D). By day 10 the cells differentiated into large, often multinucleated, and arborized cells with cytoplasmic processes (Figure 1E). The morphology was comparable to conditionally immortalized human podocytes that maintain a differentiation potential similar to their *in vivo* counterparts [21].

Conditionally immortalized human podocytes developed by transfection with the temperature sensitive *SV40-T* gene [21] are grown at permissive temperatures (33°C) to allow for cell division and at 37°C to induce cell quiescence and phenotypic changes more characteristic of mature podocytes. At 33°C immortalised podocytes underwent propagation (Figure 1F) and when incubated at 37°C displayed growth arrest with a typical arborized pattern of foot process extensions (Figure 1G). This branching of cytoplasmic processes and multinucleated morphology was also characteristic of normal human podocytes (Figure 1H) grown from outgrowths of glomeruli obtained following nephrectomy.

SEM was used to verify that iPS-derived podocytes had a typical arborized phenotype consisting of a main cell body with elongated processes extending to the periphery that is characteristic of cultured human podocytes [22], [23]. Moreover, tight junction-like structures were observed between adjacent cells (Figure 1J) and secondary and tertiary cytoplasmic processes projected from the cell bodies (Figure 1K, L).

### Characterisation of iPS-derived Podocytes

**Figure 2:**
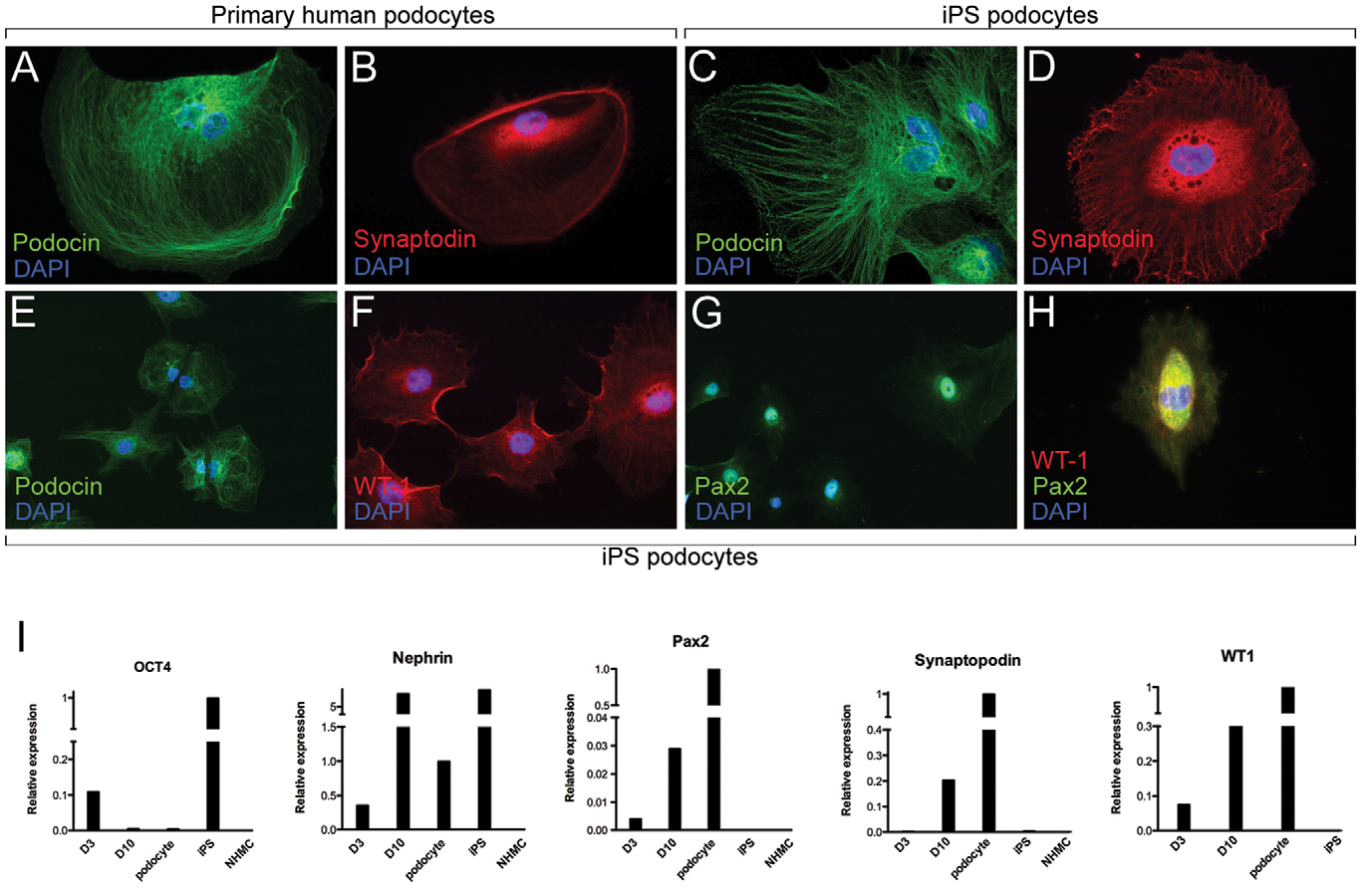
iPS Podocyte Characterisation and Gene Expression. A) Immunofluorescence microscopy showed localisation of podocin (green) in human podocytes, counterstained with DAPI. B) Human podocytes localised with synaptopodin protein (red) throughout the cytoplasm surrounding the DAPI-stained nucleus (blue). C) At 10 days of directed differentiation iPS podocytes show localisation of podocin protein, counterstained with DAPI. D) Synaptopodin (red) was expressed in the cytoplasmic matrix of iPS podocytes. E) Unlike human podocytes, iPS podocytes proliferated in culture as observed by podocin expression and DAPI (blue) and are also WT1-positive (F). Differentiated iPS cells express Pax-2 protein (G) and show nuclear co-localisation of Pax-2 and WT1 proteins (H). Mag A–D, H ×400; E–G ×200. I) qPCR showed the upregulated mRNA expression of kidney metanephric mesenchymal and podocyte genes. From day 3 (D3) to day 10 (D10) of iPS podocyte directed differentiation there was an upregulated expression of Pax2 and WT1 and podocyte-specific markers synaptopodin and nephrin at comparable levels to primary human podocytes, but different from the starting normal human mesangial cells (NHMC).

In addition to confirming morphology and growth behavior, the identity of cultured iPS podocytes was assessed by protein and mRNA expression of podocyte-specific markers using immunofluorescence microscopy and qPCR analysis. Normal cultured human podocytes showed localisation of podocin (Figure 2A) and synaptopodin (Figure 2B) protein in a filamentous arrangement, comparable to iPS podocytes differentiated for 10 days (Figure 2C). In addition, the podocyte-specific protein, synaptopodin, was localized to iPS podocytes within the extracellular matrix proteins extending towards the cell body cytoplasmic extensions (Figure 2D), as had been reported for human podocytes [21]. Conversely, unlike human podocytes that show reduced metabolic activity reaching quiescence in culture [24], iPS podocytes proliferate (Figure 2E, F) and could be maintained up to the 3 months analysed. Further, they showed a co-localised nuclear expression of Pax-2 and WT1 (Figure 2 G–H).

By qPCR, undifferentiated iPS cells expressed OCT3/4 that was downregulated over the timecourse of directed differentiation. By 10 days of culture, the differentiated iPS cells expressed OCT3/4 at baseline levels comparable to both human podocytes and the starting human mesangial cells prior to reprogramming (Figure 2I). Conversely, markers characteristic of renal progenitors in the metanephric mesenchyme, namely WT1 and Pax-2 were upregulated 4-fold and 7-fold, respectively, relative to the expression in cultured human podocytes. In addition, differentiated iPS-derived podocytes after 10 days showed a 24-fold and 7-fold upregulated expression of the podocyte-specific genes synaptopodin and nephrin, respectively. The starting population of human mesangial cells were negative for expression of the genes analysed. Undifferentiated iPS showed a characteristic positive expression for nephrin. However, nephrin mRNA expression decreased following early initiation of directed differentiation (day 3) but then further increased with cell maturation over time of culture (day 10), comparable to other mesenchymal and podocyte genes.

### Functional Analysis

**Figure 3:**
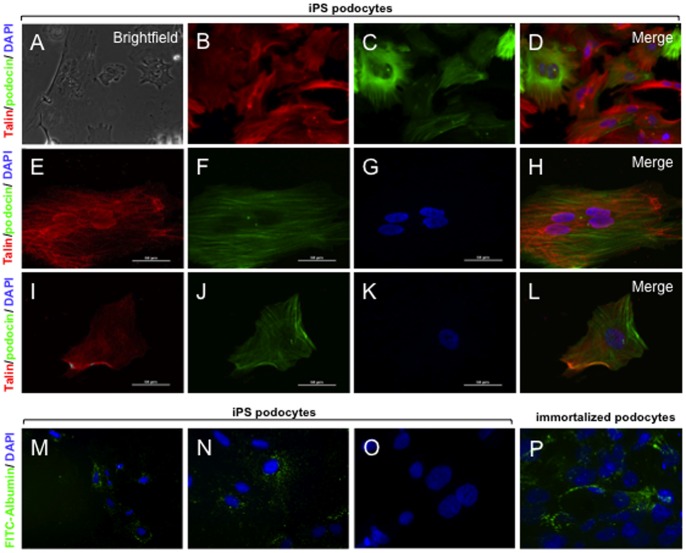
Functional contractility and permeability. Live cell imaging was used to record the response of iPS podocytes to the addition of AII (See Movie S1). A) Phase contrast imaging of iPS podocytes following the addition of AII at time 0. The iPS podocytes were transduced with RFP-actin (B) and immunostained with the contractile protein, podocin (C). D) Merge image of actin (red) and podocin (green) with DAPI-stained nuclei (blue). E) Confocal immunofluorescence shows that iPS-podocytes transduced with RFP-talin (E) co-expressed podocin (F) at time 0. G) DAPI-stained nuclei and merged image are shown (H). After 6 hours in culture, the iPS podocytes were viable and display a contracted morphology in response to AII (I–L) where RFP-talin (red), podocin (green) and DAPI (blue) are shown. (M-N) By fluorescence microscopy, the iPS podocytes were able to uptake FITC-albumin (green) into the cytoplasm when cultured at 37°C, compared to iPS podocytes cultured at 4°C (O) that served as a control. (P) Immortalized human podocytes also showed endocytosis of FITC-albumin in a similar morphological pattern. Mag A–D ×200; E–L, N-P ×1000; M ×100. Abbreviations: angiotensin II (AII); red fluorescent protein (RFP).

Functional assays for podocytes included a contractile response to the addition of AII and the uptake of albumin. Podocytes and cytoplasmic foot process extensions develop an actin-based contractile system that are distributed in long bundles with other scaffolding proteins including talin [24] that contract in response to AII. Under phase contrast microscopy the dynamic changes in cell morphology were observed by time-lapse imaging (Figure 3A; Supplementary Figure 1). Using immunofluorescence microscopy, differentiated iPS podocytes showed a cytoplasmic distribution visualised with baculoviral transduced red fluorescent protein (RFP)-labelled actin (Figure 3B–D) and talin (Figure 3E–L) that was prominently localised adjacent to podocin. Confocal immunofluorescence microscopy showed that the addition of 500 nM of AII induced an initial contraction of the iPS podocyte cell body and shortening of the cytoplasmic processes in a time-course dependent characteristic of de-differentiating podocytes [25].

A permeability assay was used to determine the endocytic uptake of FITC-labeled albumin as further evidence of podocyte-like functional characteristics. By fluorescence microscopy FITC-albumin uptake by iPS podocytes was time- and temperature-dependent in iPS podocytes differentiated for 10 days. An uptake of FITC-albumin in the cytoplasm of iPS podocytes (Figure 3M, N) was distinct compared to control iPS podocytes cultured at 4°C that did not show endocytic incorporation of albumin (Figure 3O). This pattern of cytoplasmic uptake of FITC-albumin in iPS podocytes (Figure 3P), was similar in pattern to the endocytosis of albumin previously observed in immortalized human podocytes [26].

### *In vitro* Integration of kiPS-derived Podocyte Progenitors

**Figure 4:**
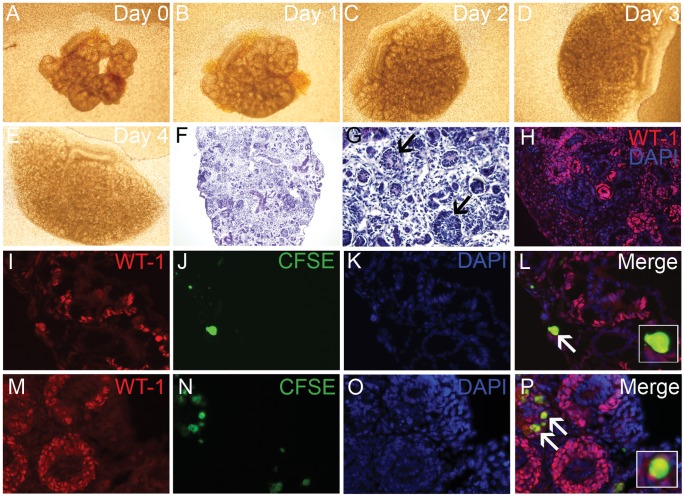
iPS podocyte integration into embryonic kidneys. A–E) Bright field microscopy showing representative E13.5 mouse embryonic kidneys (n = 3) reaggregated with CFSE-labeled iPS podocytes and grown as an explant over 4 days in culture. Histology showed that the dissociated kidneys explants successfully reaggregated (F) that displayed definitive features including developing glomeruli (G; *arrows*) and tubular segments. H) Immunofluorescence microscopy showed WT1 (red) cells in glomerular aggregates with DAPI-stained nuclei (blue). I-L) Using immunofluorescence of WT1 protein (I; red), a co-localised expression was observed in CFSE-labeled iPS podocytes (J; green) counterstained with DAPI (K). The iPS cells were observed within glomerular aggregates of E13.5 kidney explants (Merge; L). M-P) E15.5 explants immunostained with WT1 (M; red) and CFSE-labeled iPS podocytes (N; green) counterstained with DAPI (O) showed further integration into WT1-positive glomerular structures (Merge; P). Counterstaining with DAPI (O). Mag A–E ×4; F ×100; G ×200; H ×200; I–P × 400.

An *in vitro* nephrogenesis reaggregation assay was developed to investigate the differentiation and integration capacity of iPS-derived podocyte progenitors following interaction with the microenvironmental cues of the developing kidney. Adapting the technique of an embryonic kidney reaggregation assay [27], differentiated CFSE-labeled iPS podocytes were reaggregated with partially dissociated E13.5–15.5 embryonic kidneys and grown as an explant over four days of culture (Figure 4 A–E). The reaggregation of 30,000 iPS differentiated podocytes with 2 or 3 E13.5 or E15.5 embryonic kidneys, respectively, allowed for a large number of viable CFSE+ cells to remain after 4 days in culture. Within the tissue reaggregate the cells in close association with the regions of nephrogenesis showed organisation into nephron structures including WT1-positive induced metanephric mesenchyme and developing glomeruli (Figure 4F–H).

The incorporation of CFSE-labeled iPS podocytes into WT1 positive glomerular aggregates was assessed using developing kidneys that were reaggregated and cultured as explants (Figure 4 I–P). The CFSE-labeled differentiated iPS podocytes (10 days) were viable and located in the outer region of the kidney where they were shown to co-express WT1 protein using immunofluorescence labeling (inset; Figure 4L, P). The co-expression and integration of iPS podocytes into WT1-positve glomerular aggregates were more frequent in the mature E15.5 kidney reaggregation kidney explants when glomerular development was more established (Figure M–P). In sharp comparison, no integration of CFSE-labeled immortalized podocytes was evident when reaggregated with embryonic kidney explants.

## Discussion

The progression of glomerular disorders leading to fibrosis and loss of function is dependent on the severity of podocyte injury and the capacity for tissue regeneration and podocyte replacement. The differentiation of putative progenitor cells derived from the glomerular parietal epithelium of the Bowman’s capsule may provide a source of replacement cells during normal glomerular homeostasis [28], [29], [30]. Developmental pathways such as Notch [31] and Wnt-β-catenin [32] may partially regulate podocyte differentiation and/or renewal that play important roles in governing outcome of regeneration or the development of disease. In a mature differentiated state, human podocytes expresses proteins such as WT1, nephrin, synaptopodin and podocin [20]. However, the propensity of podocytes to rapidly differentiate in culture and enter irreversible growth arrest has made long-term propagation difficult.

The loss of expression of podocyte specific proteins correlates with de-differentiation, both in culture, and as a hallmark of glomerular disease [33], [34]. The directed differentiation of iPS generated cells that expressed markers of induced metanephric mesenchyme, including WT1 and Pax-2, in addition to specific markers of mature podocytes. Mature podocytes that present cell cycle arrest and become quiescent are slow growing and difficult to propagate in culture. In comparison, the iPS-derived cells retained a proliferative potential long-term following directed differentiation. This was achieved by the removal of activin A, BMP7, and retinoic acid from the culture medium after 10 days, however while retaining the phenotypic expression of podocyte markers in the iPS-derived podocytes.

The induced expression of WT1 target genes in podocyte precursors may specifically activate cell cycle regulation leading to the induction of mature podocyte markers [35]. Pax-2 co-expression with WT1 has been reported in normal human parietal podocytes that also express mature podocyte markers, and may signify that these cells have retained the ability to divide [36]. Furthermore, the re-expression of Pax-2 in conjunction with WT1 in human podocytes may serve as a forerunner to a recapitulation of developmental paradigms leading to podocyte de-differentiation in glomerulosclerosis [37]. Mature podocytes also express key components of the renin-angiotensin system, and respond to TGF-β [38], [39]. These pro-inflammatory mediators of fibrosis can directly affect the podocyte leading to foot process contraction and podocyte effacement. In response to TGF-β and AII, mature podocytes undergo de-differentiation [25], [40]. The resulting flattening and contraction of cytoplasmic processes was also observed in the iPS-derived podocytes in response to AII and was associated with shortening of the extracellular matrix proteins actin and talin.

There are many advantages of developing iPS cells as an investigative strategy for patients with genetic and non-genetic kidney disease that will provide a valuable tool to culture and manipulate podocytes *in vitro* in order to understand their biology and model human disease. We have previously reported that iPS cells can be derived from human mesangial cells [17]. In addition, epithelial cells obtained from the urine of kidney disease patients may offer an obtainable source of kidney cells for generation of iPS [18]. Several studies have reported genetic and epigenetic transcriptional variation between iPS cultures [41], [42], [43] where the cell type of origin may influence the capacity for *in vitro* differentiation potential [44]. Indeed, DNA methylation states change following directed differentiation, however aberrations in epigenetic imprints are frequent in iPS colonies following cell reprogramming where the epigenetic instabilities may persist [45]. Therefore, for future consideration transcriptional-based direct reprogramming of kidney cells using a strategy of re-expressing key developmental regulators may offer an alternative, as has been shown in neurons [46], [47], cardiomyocytes [48] and β-islet cells [49].

In the interim, the directed differentiation of iPS cells to podocytes offers an unprecedented opportunity to generate human cells that represent kidney disorders and divide long-term, thereby enabling disease investigation, drug development and disease-modifying assays. The screening of genetic mutations in disease-derived iPS cells and the development of cellular assays will also serve as a fundamental step for future studies to correct the genetic defects in podocyte progenitors that maintain a proliferative capacity. The generation of podocytes with a corrected version of the defective genes may offer an avenue for cellular replacement in glomerulopathies such as congenital nephrotic syndrome and hereditary forms of focal and segmental glomerulosclerosis (FSGS). This selective approach may provide an avenue for isolating isogenic and functionally restored iPS cells. In this regard, the autologous transplantation of iPS podocytes with correction of the genetic defect may have a profound ability to alter disease progression and will also allow for the examination of how the mutations underlying kidney disease affect podocytes at a cellular level.

## Concise Methods

### Retroviral Transfection of Human Mesangial Cells

iPS cells were derived from normal human kidney mesangial cells (NHMC; Lonza) using retroviral transfection of *OCT3/4, SOX2, KLF4* and *c-Myc* as previously described [17]. The NHMCs were cultured in MsGM Mesangial Growth Media with 5% fetal bovine serum (FBS) at 37°C. Retroviruses containing human *OCT3/4, SOX2, KLF4 and c-Myc* were introduced into 293FT cells and the supernatant cultured with NHMCs for 24 hours. After 5 days, the transduced NHMCs were reseeded onto MEF feeders in KO DMEM (Gibco Invitrogen, CA, USA) containing 20% KO (knockout) serum replacement and human basic FGF (10 ng/ml). Eighteen days after transduction, colonies were mechanically dissociated for re-plating. The kidney-derived iPS cells were pluripotent, showed a normal karyotype and exhibited silencing of the retroviral transgenes after passage four of differentiation [17].

### Differentiation of iPS Cells to Podocyte Progenitors

The iPS colonies were mechanically cut into small pieces approximately the same size, and were cultured in ultra low cluster 6-well plate (COSTAR) for 3 days in the differentiation medium consisting of DMEM–F12 (Sigma) with 2.5% FBS, 100 µM nonessential amino acids, 100 µM beta mercaptoethanol with the addition of 10 ng/ml of activin A, 15 ng/ml of BMP7, and 0.1 µM retinoic acid. The cells were transferred into 0.1% gelatin pre-coated 10 cm tissue culture dishes for another 7–8 days in the same medium before serial sub-passaging for characterization and integration assays. At 10 days of differentiation the iPS podocytes were fixed in 2.5% glutaraldehyde in cacodylate buffer and processed for SEM visualization (Hitashi S570 microscope). For the long-term maintenance of iPS podocytes, after 10 days of directed differentiation the iPS-derived podocytes were grown in DMEM-F12 media without the addition of 10 ng/ml of Activin A, 15 ng/ml of BMP7, and 0.1 µM retinoic acid where they were able to maintain the morphological characteristics and functional capacity.

### Isolation and Labeling of Human Podocytes

Human kidneys were obtained from patients scheduled for nephrectomy following written consent and approval from the Southern Health Human Ethics Committee (approval #10179B), Monash Medical Centre. Human podocytes were derived from normal kidney tissue using a sieving method adapted from isolation of mesangial cells [50]. Normal kidney tissue was minced and passed through two mesh sieves (120 and 105μ) using a series of washes, centrifugation, syringe dissociation and resuspension in DMEM-F12 media. Decapsulated glomeruli were grown as explants for approximately 30 days. Human podocyte outgrowths were easily identified by morphology and subsequently sub-passaged and plated into chamber slides for immunofluorescence staining and RNA extraction. The human podocytes were confirmed to show positive protein and gene expression for podocyte-specific markers podocin, synaptopodin and nephrin.

Immortalised podocytes grown at 33°C and 37°C and iPS-derived podocytes were labelled using carboxyfluorescein diacetate succinimidyl ester (CFSE; Life Technologies, Australia). Briefly, cells were harvested, counted and incubated with 15 µM CFSE for 15 minutes at 37°C before centrifugation and washing in media. To ensure complete integration of the CFSE probe the cells were further incubated for 30 minutes at 37°C, washed with PBS and resuspended in DMEM containing 10% FBS, 1% Pen/Strep, 1% L-Glutamine and 1% Insulin Transferrin Selenium (Life Technologies).

### Immunofluorescence Microscopy

Immunocytochemistry was performed as described previously [17]. Differentiated iPS podocytes and human podocytes seeded on chamber slides in 10% FBS medium were serum-starved overnight, then fixed in 4% paraformaldehyde (PFA) for 10 min, permeablized with 0.1% Triton X-100/PBS for 10 min and incubated in blocking solution (4% normal goat serum/PBS) for 30 min. The cells were incubated with anti-nephrin, anti-synaptopodin, anti-Pax-2, and anti-podocin (NPHS2; all from Abcam, USA) and anti-WT1 (Santa Cruz, USA) at dilutions from 1∶20–400 in blocking solution overnight at 4°C and incubated with secondary antibodies (Alexa Fluor 488, 555 dilution 1∶1000) in PBS for 1 hr. Sections were counterstained with DAPI (1∶10,000; Life Technologies) and then mounted with Fluorescent Mounting Medium (DakoCytomation, Denmark) and analysed with a Provis AX70 (Olympus, Japan) or Nikon C1 confocal fluorescent microscope (Nikon, Japan).

### qPCR

Total RNA was extracted from undifferentiated iPS cells, normal human podocytes obtained from primary culture, and iPS podocytes at day 3 and day 10 of directed differentiation using a pico pure RNA isolation kit (ARCTURUS). cDNA was synthesized using SuperScript III first–strand synthesis system for TR-PCR (Invitrogen). Quantitative real-time PCR (qPCR) was performed using a platinum SYBR Green qPCR SuperMix-UDG (Invitrogen). The threshold cycle (Ct) values were measured in triplicate and normalized against the endogenous control β-actin and expression levels normalized against β-actin using primers listed in Figure S1.

### Cell Contractility and Permeability

iPS podocytes at a seeding density of 0.5×10^4^ cells/well were transduced using a Cell Lights™ (Life Technologies) intracellular RFP-actin and RFP-talin. Cells were incubated with Cell Lights™ 2.0 reagent overnight before replacing with serum-free DMEM media for 2 hours. The iPS podocytes were visualised every 15 minutes with/without the addition of AII (500 nM) using a Leica AF6000 LX (Leica, Germany) live cell imaging system. Following 24 hours of live cell imaging (Movie S1) the control and AII-treated iPS podocytes were fixed with 4% PFA and immunostained with anti-podocin antibody before visualisation with a Nikon C1 confocal microscope.

For the permeability assay, differentiated iPS podocytes were cultured as described above, and, after differentiation the culture medium was replaced with serum-free media with/without FITC-labeled albumin (0.5 mg/ml; Abcam) and cultured at 37°C for 1 hour, in comparison to control cells cultured at 4°C. The cells were fixed in 4% paraformaldehyde (PFA) and counterstained with DAPI. Assays were performed in triplicate using separate cell preparations.

### Reaggregation Assay

All animal experiments were approved in advance by a Monash University Animal Ethics Committee, which adheres to the “Australian Code of Practice for the Care and Use of Animals for Scientific Purposes. Embryonic day (E) 13.5–15.5 embryos were collected from time-mated pregnant C57BL6/J mice. For the explants, 2 (E15.5) or 3 (E13.5) embryonic kidneys were transferred into Eppendorf tubes containing 30,000 CFSE-labelled cells and gently mechanically disrupted using a 25 G needle before centrifugation (3 minutes, 18 rcf) to form aggregates that were transferred onto a floating polycarbonate membrane (3 µm pore size; GE Water & Process Technologies, Australia) in DMEM growth medium in a 24 well plate. The aggregates were incubated (5% CO_2_, 37°C) for 4 days with a medium change after 48 hours. Photomicrographs were taken daily to assess aggregate growth (Olympus IX51 dissecting microscope). After 4 days of culture, the kidney explants were fixed with 4% PFA for histological examination with hematoxylin and eosin staining. CFSE-labeled cells were visualised by immunofluorescence microscopy of frozen sections (5 µm) that were immunostained with WT1 and counterstained with DAPI. Embryonic kidneys reaggregated with CFSE-labeled immortalized podocytes were cultured over the same time period as a control comparison.

## Supporting Information

Figure S1 **Table detailing the primers used for real-time quantitative PCR.**
(DOC)

Movie S1 **Phase contrast microscopy using time-lapse imaging shows the dynamic changes in cell morphology following the addition of 500 nM of angiotensin II.** Over 24 hours, the differentiated iPS podocytes showed a contractile response and retraction of cytoplasmic processes following the addition of angiotensin II, however without affecting cell viability.
(MOV)
