# Supplementary material for: The Directed Differentiation of Human iPS Cells into Kidney Podocytes
Source: PLoS One. 2012 Sep 28;7(9):e46453. doi: 10.1371/journal.pone.0046453 (PMC3460883; doi:10.1371/journal.pone.0046453)
Supplement: Figure S1 — Table detailing the primers used for real-time quantitative PCR. (DOC) [file pone.0046453.s001.doc]

**Supplementary Figure 2**

**Primers for real-time PCR**

| Gene name | forward | reverse |
| --- | --- | --- |
| **WT-1** | GGACAGAAGGGCAGAGCAACCA | GTCTCAGATGCCGACCGTACAA |
| **Synaptopodin** | AGCCCAAGGTGACCCCGAAT | CCCTGTCACGAGGTGCTGGC |
| **PAX2** | AACGACAGAACCCGACTATG | ATCCCACTGGGTCATTGGAG |
| **Nephrin** | CAACTGGGAGAGACTGGGAGAA | AATCTGACAACAAGACGGAGCA |
| **OCT4** | GACAGGGGGAGGGGAGGAGCTAGG | CTTCCCTCCAACCAGTTGCCCCAAAC |
| **Actin** | GTGGGCATGGGTCAGAAGGA | CTCGCGGTTGGCCTTGGG |
